# A Naturalistic Study of the Maintenance of Gains Made With Treatment of Patients With Profound Treatment-Refractory Obsessive-Compulsive Disorder

**DOI:** 10.3389/fpsyt.2021.673390

**Published:** 2021-07-20

**Authors:** Nighat Jahan Nadeem, Emily Chan, Lynne Drummond

**Affiliations:** ^1^South West London and St George's Mental Health NHS Trust, London, United Kingdom; ^2^St George's University of London, London, United Kingdom

**Keywords:** obsessive-compulsive disorder, inpatient, treatment gains, maintenance, follow up after discharge

## Abstract

Obsessive-compulsive disorder (OCD) generally responds to first-line treatment but patients often relapse. The United Kingdom National OCD Inpatient Service treats patients who have failed to respond to at least two trials of SRI, augmented with a dopamine blocker and two trials of ERP. Despite this, they have profound treatment-refractory OCD and require 24-h nursing care due to severe OCD. We examined patients' Y-BOCS score on admission, discharge and at each follow-up from all patients discharged over 5 years (02/01/2014-31/12/18). Data were analysed using SPSS. Paired student *t*-tests were used to assess improvement from admission to discharge and each follow-up. Over 5 years, 130 adult patients were treated: 79 male and 51 female with an average age of 42.3 years (20-82; sd14.4). Their ethnic backgrounds were; 115 Caucasian, 11 South Asian, 1 Chinese, and 3 Unspecified. On admission, the average Y-BOCS total score was 36.9 (30-40; sd2.6). At discharge, patients had improved on average by 36% (Y-BOCS reduction to 23.4 = moderate OCD). Similar reduction in Y-BOCS continued throughout the year with an average Y-BOCS of 22.9 at 1 month (*n* = 69); 23 at 3 months (*n* = 70); 21.3 at 6 months (*n* = 78) and 21.9 at 1 year (*n* = 77). Twenty-seven patients did not attend any follow-up appointment whilst others attended at least one appointment with the majority attending more than 3. Using student *t*-test, improvements at discharge, 1, 3, 6, and 12 months post-treatment showed a highly significant improvement (*p* < 0.001). Gains made following inpatient treatment for treatment-refractory OCD were generally maintained until 1 year post-treatment.

## Background

Treatment for obsessive-compulsive disorder (OCD) consists of psychopharmacological agents which act on the serotonin system and psychological treatments involving exposure and response prevention (ERP). However, approximately one third of patients do not respond to first line pharmacological treatment (1) and despite the efficacy of cognitive and behavioural interventions, they are only effective in 50-60% of cases, with as few as 25% experiencing full recovery (2). The definition of what constitutes a patient with refractory OCD has varied in the literature. Some authors have described them as having failed to respond to trials of adequate dosages of more than one serotonin reuptake inhibitor (SRI) (3), whilst others have included failure to respond to at least two trials of SRIs in addition to ERP (4).

In addition to the fact that a high proportion of people treated for OCD fail to derive benefit, there has also been evidence that up to 50% of patients relapse after treatment (5–7). On the other hand, another study found that 2 years after treatment with group cognitive-behavioural therapy (CBT), 78.6% of patients remained in remission; in this study the patients had severe OCD with a mean baseline score on the Yale Brown Obsessive Compulsive (Y-BOCS) Scale of 25.3 (severe OCD) (8).

In the UK in 2005, the National Institute for Health and Clinical Excellence (NIHCE) published guidance about the recommended treatment for OCD which sets out first line and second line treatments including psychopharmacological and psychological interventions (9). The approach to the treatment of OCD is described as a stepped-care model with Tiers 1 through to 5. In Tier 5, patients are profoundly ill having failed to respond to all previous treatments. It is recommended that these patients are treated in highly specialised teams with extensive expertise in the treatment and management of OCD.

In response to the 2005 NIHCE Guidance, the National Department of Health funded highly specialised teams to treat the most profoundly ill patients with OCD who had failed to respond to all previous treatments (10). In order to be eligible for treatment in one of the highly specialised services, patients have to:

1. Score greater than 30/40 on Y-BOCS.
2. Have received at least two previous trials of SRIs in maximum licenced dosages for a minimum of 3 months each and without response.
3. Have had at least one of those trials of SRIs augmented in a way recommended by Pallanti et al. This most commonly was augmentation with a dopamine blocker (11).
4. Have received two trials of CBT incorporating ERP where one of these trials should have taken place in the patient's own home or in whichever environment the symptoms are maximal.

Patients met the threshold for inpatient treatment if they failed to improve with the above interventions. Patients were also eligible if they were a risk to themselves because of self-neglect related to their OCD or if they had other difficulties such as urinary or faecal incontinence.

The service at South West London and St George's Mental Health NHS Trust comprises the only 24 h staffed dedicated inpatient service for OCD patients in UK that is funded centrally. Data from this Service demonstrates that inpatient care is effective and patients benefit from a 40% reduction in OCD symptoms; these gains are generally maintained on average at 19 months follow-up (12, 13). Patients are cared for by specialised nurses and given individualised treatment consisting of psychological and pharmacological interventions (14). Our patients are encouraged to remain on their prescribed medication (SRIs in particular) and the evidence is that SRIs are required for the long term treatment of severe OCD. It is thought that SRIs are effective in both maintenance treatment as well as to prevent relapse (15). Several factors have been identified as predictive of increased likelihood of relapse, including not having CBT in the interval period, poorer quality of life at baseline, shorter duration of follow-up and later age at onset (16).

Evidence suggests that the combination of medication and psychological therapy is effective in OCD and one study found a 41% reduction in total Y-BOCS score after treatment with CBT + SRI/placebo and this improvement was sustained after 6-8 years (17). This study also suggested that patients may benefit from ongoing psychological treatment post-discharge. A recent systematic review and meta-analysis of 36 trials investigating the effect of CBT with ERP in OCD has highlighted several concerns with methodological rigour and issues with such studies including the risk of bias, treatment fidelity and the impact of researcher allegiance (18). Despite reported improvements in treatment of OCD with pharmacological and/or psychological interventions, there are few long-term studies of profoundly ill OCD patients who have received pharmacotherapy and CBT involving ERP. We decided to examine outcome in all patients discharged from our ward over a 5 year period and investigated the maintenance of gains over the first year post-discharge.

## Materials and Methods

In this naturalistic study, we analysed data from the electronic medical records of all patients who had been discharged from our service in a 5 year period from 02/01/2014-31/12/18.

Patients who had been unable to attend the hospital for assessment had been assessed *via* telephone and where necessary thereafter this was followed up by a home visit irrespective of the distance they lived from the hospital. Patients who were unable to accept inpatient treatment were offered home-based therapy with the aim of overcoming the obstacles to their accepting admission. For those patients who agreed to be admitted to the unit, all had received multiple trials of pharmacotherapy and so on admission, the response to each medication was discussed with the patient and, in collaboration with them an optimal regime was discussed. For some patients this meant remaining on the regime they had been taking already and for others switching medication.

The basis of the therapy regime with the inpatients was a therapy session at least weekly with a therapist as well as daily sessions with the nurses. This included behavioural interventions of graded exposure with self-imposed response prevention. Although there were dedicated CBT therapists, many members of the inpatient team had qualifications in CBT and this included senior medical and some nursing staff. Daily group and individual sessions were provided by the occupational therapist. These sessions were also based on the concepts of graded exposure and response prevention and were created individually with each of the patients. Following inpatient treatment, all patients were encouraged to create their own individualised relapse prevention plan which was also shared with their local team. The National Specialist Unit routinely follows up the patients at 1 week; 1 month; 3 month; 6 months and 1 year after the inpatient stay by a member of the clinical staff either remotely or in person. The severity of OCD symptoms were measured as part of the follow up using the Y-BOCS scale. Y-BOCS total scores on admission and discharge as well as at each follow up were extracted and analysed. As patients did not attend every follow-up, numbers vary over the year. Basic demographics including age, gender and ethnicity were also extracted and data were analysed using SPSS software. Paired student *t*-tests were used to evaluate improvement in Y-BOCS scores from admission to discharge and each follow up appointment and using intention to treat analyses.

## Results

Over 5 years, 130 patients were treated; 79 men and 51 women with an average age of 42.3 years (20-82; sd14.4). Their ethnic backgrounds were; 115 Caucasian, 11 South Asian, 1 Chinese, and 3 Unspecified.

**Figure 1:**
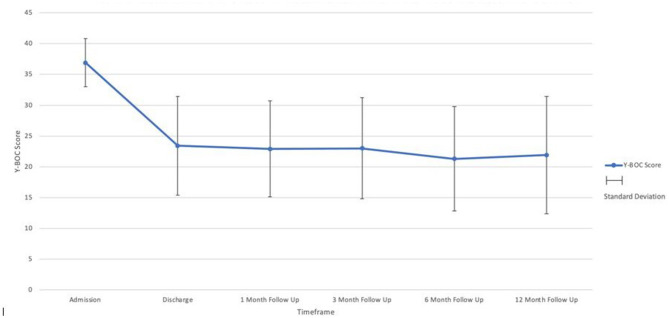
OCD severity as indicated by the Y-BOCS total at admission, discharge, and respective follow-up points.

**Figure 2:**
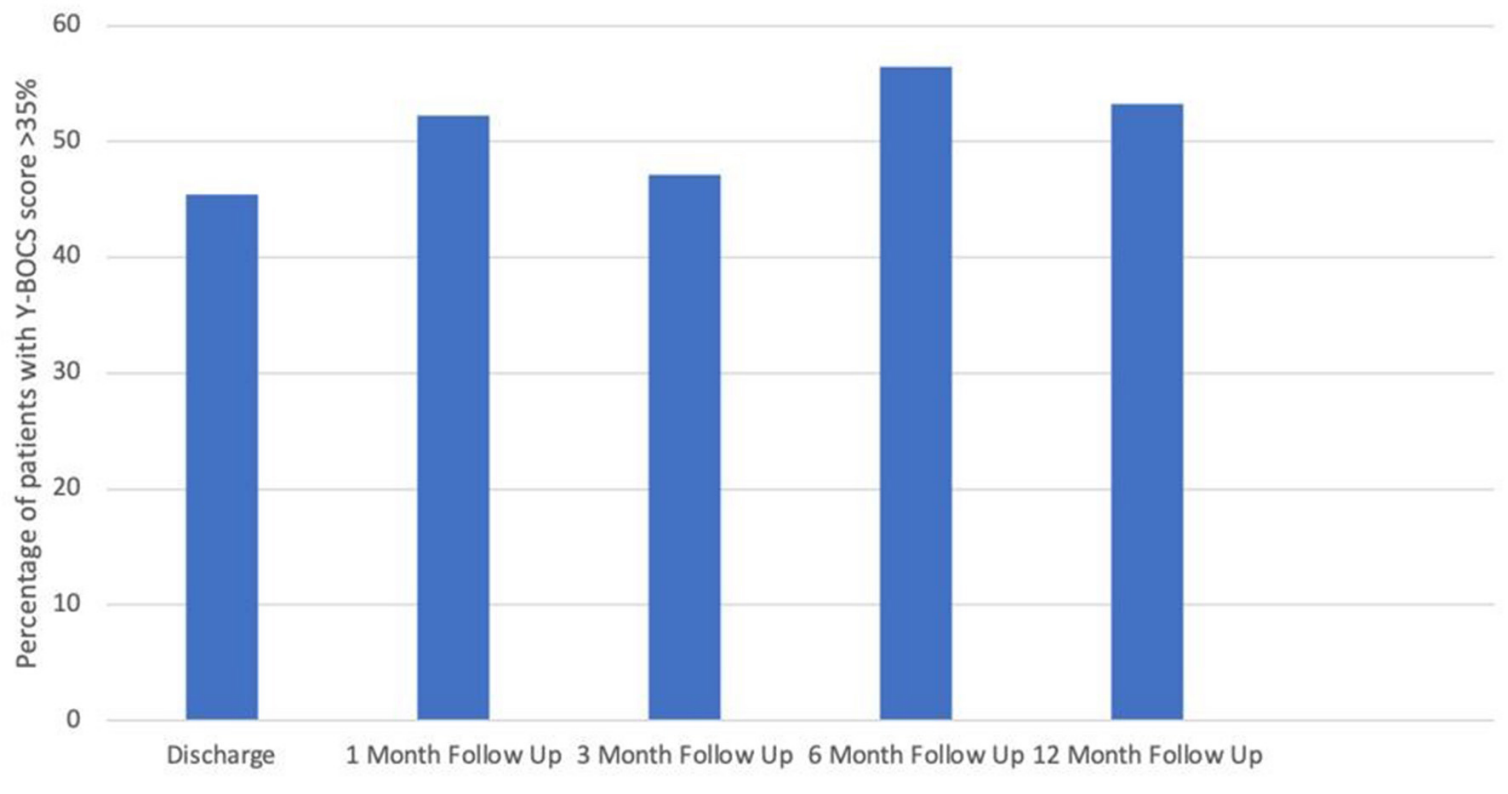
Percentage of patients with >35% improvement in Y-BOCS score at each follow up point (compared with admission Y-BOCS score).

On admission, the average Y-BOCS score was 36.9 (30-40; sd2.6). At discharge, patients had improved on average by 36% (Y-BOCS reduction on average to 23.4 = moderate OCD). Similar reduction in Y-BOCS continued throughout the year with an average Y-BOCS of 22.9 at 1 month (*n* = 69); 23 at 3 months (*n* = 70); 21.3 at 6 months (*n* = 78) and 21.9 at 1 year (*n* = 77) (Figures 1, 2).

Twenty seven patients did not attend any follow-up appointment whilst others attended at least one appointment with the majority attending more than 3. The reasons for loss to follow up were not explored in this study and most of these patients were those who had failed to derive benefit from their inpatient stay.

Using paired *t*-test; improvements at discharge, 1, 3, 6, and 12 months all showed a highly significant improvement (*p* < 0.001).

## Discussion

This study adds to the existing evidence of the longer term impact of combined treatment (medication with CBT/ERP) in patients with treatment-refractory OCD Patients with profound treatment-refractory OCD had a 36% reduction in their Y-BOCS scores following inpatient treatment. This improvement from admission to discharge reflects an improvement from profound treatment-refractory OCD to moderate OCD. This improvement is sustained over the first year post-discharge from the ward with minimal fluctuations in their Y-BOCS scores during this period.

Previous studies have explored the impact of either pharmacological or psychological treatments on OCD. During their inpatient stay our patients received a combination of both psychopharmacology which was optimised on admission and CBT with ERP and we assessed the longer term (over 1 year) impact of this combined treatment approach. Many studies have assessed the impact of OCD treatment in outpatients with moderate-severe OCD. Similar studies conducted on both inpatient and outpatient samples have demonstrated relapse estimates of up to 50% (5–7). Our findings highlight the long term benefits of intensive inpatient treatment in patients with profound treatment-refractory OCD and adds to existing evidence which highlighted the benefits of treatment with a 40% reduction in symptoms which is maintained in the long term after discharge from the national service in the United Kingdom (12).

Our service provides individualised treatment programmes including medicine optimisation, creating individualised exposure programmes combined with group occupational therapy sessions focussing on facing up to fear and activities of daily living. As such this could be replicated elsewhere. The importance of a dedicated service where patients can advise, inspire and help each other should not be underestimated.

In our study, patient adherence to follow up appointments was good with 60% attending three or more appointments after discharge. However, we were unable to gather follow up data for ~20% of the patients we treated over the 5-year period. A further study exploring reasons for lack of follow up in this subgroup of patients would be beneficial to improve our understanding of the longer term impact of inpatient OCD treatment.

This study did not take into account other measures of OCD not listed in the Y-BOCS or other factors influencing a patient's recovery such as time spent in inpatient service, quality of life, comorbid mental health problems or use of other psychotropic medication. It is unusual for a patient admitted to the service not to also have clinical signs of depression and previous studies from this unit have demonstrated that almost 80% of patients admitted had clinical evidence of moderate or severe depression as indicated by the Beck Depression Inventory (19). A retrospective study of patients admitted to this service found that 21% of the sample had autistic spectrum disorder; 12.4% had emotionally unstable personality disorder and 18.5% had obsessive-compulsive personality disorder (20). It would be useful to explore the impact of such comorbid factors in the longer term after discharge from the ward. Overall, this study demonstrates that intensive inpatient treatment on the National OCD Unit has a lasting and sustained positive impact for at least 1 year post-discharge.

### Strengths

This was a naturalistic study analysing data from patients with profound treatment-refractory OCD and this is one of the few studies to date of long term follow up post discharge after inpatient OCD treatment. This information fills an important knowledge gap in the literature.

All patients admitted to and discharged from the unit over a 5 year period were followed up for 1 year post discharge providing data over a significant time period which is difficult to collect.

This provides a robust sample size of 130 patients who were all treated at the same centre which adds to the validity of the results.

The same outcome measure (Y-BOCS total score) was used at each follow up to ensure that the data is comparable with minimal confounding variables.

Data was sourced from patients' electronic records ensuring the use of accurate and reliable information as documented by clinicians.

### Limitations

Although this study explored long term patient outcome after inpatient treatment, only the Y-BOCS score was used to determine patients' symptom severity. No other follow-up data was assessed. This study was not designed to assess other measures and data was based on convenience sampling of existing clinical data.

A minority of patients were lost to follow up due to unknown reasons. Further information related to this would be important as well as ongoing medication use.

Any comorbidities patients had were not assessed or monitored in this study.

### Implications for Practise

This study demonstrates that a majority of patients with the most profound refractory OCD are able to maintain the gains made for at least 1 year after treatment with optimised psychopharmacology as well as CBT with ERP. It would be beneficial to further study whether the benefit of treatment continues to be sustained after 1 year post discharge.

## Conclusion

This study demonstrates evidence that the gains made following inpatient treatment for treatment-refractory OCD are generally maintained until 1 year post-treatment.

## Data Availability Statement

The original contributions presented in the study are included in the article/supplementary material, further inquiries can be directed to the corresponding author/s.

## Author Contributions

NN and LD designed the study, analysed the results, and wrote the paper. EC completed data extraction. All authors contributed to the article and approved the submitted version.

## Conflict of Interest

The authors declare that the research was conducted in the absence of any commercial or financial relationships that could be construed as a potential conflict of interest. The handling editor declared a past co-authorship with one of the authors LD.
